# Adverse Drug Effect Profiles of Gp2b/3a Inhibitors: A Comparative Review of the Last Two Decades

**DOI:** 10.7759/cureus.49332

**Published:** 2023-11-24

**Authors:** Naziha Hasan, Walter Jauregui, Mahrukh Zubair, Venugopala K Pushparajan, Bryan J Carson, Durga Manaswini Attaluri, Diny Dixon, Aman Jaisinghani, Andres Chuecos, Deepika Ravichandran

**Affiliations:** 1 Emergency Department, Derriford Hospital, Plymouth, GBR; 2 General Medicine, Universidad Nacional Autónoma de Honduras, Tegucigalpa, HND; 3 General Medicine, Mohi-ud-Din Islamic Medical College, Azad Jammu Kashmir, PAK; 4 General Medicine, Vydehi Institute of Medical Sciences and Research Centre, Bengaluru, IND; 5 Emergency Medicine, Northern Health and Social Care Trust, Coleraine, GBR; 6 Critical Care, Sri Aurobindo Institute of Medical Sciences, Indore, IND; 7 General Medicine, Jubilee Mission Medical College and Research Institute, Thrissur, IND; 8 Emergency Medicine, Ziauddin University Hospital, Karachi, PAK; 9 General Medicine, La Universidad de los Andes, Mérida, VEN; 10 General Medicine, American University of Antigua College of Medicine, St John’s, ATG

**Keywords:** acute chest syndrome (acs), pubmed, comparative review, orofiban, eptifibatide, tirofiban, abciximab, nstemi, stemi, gp2b/3a inhibitors

## Abstract

ST-Elevation Myocardial Infarction and non-ST Elevation Myocardial Infarction belong to the acute coronary syndrome group of diseases. These conditions are characterized by the complete or partial blockage of one or several coronary arteries, resulting in myocardial injury or necrosis. Various medications are used in their treatment, with the most recent addition being Glycoprotein IIb/IIIa inhibitors. They work by hindering the activity of glycoprotein IIb/IIIa receptors, which, in turn, prevents the clumping of platelets. Some of the GpIIb/IIIa inhibitors available in this category include abciximab, tirofiban, eptifibatide, roxifiban, and orbofiban. With this comprehensive literature review, we aimed to explore the potential adverse effects of these medications and compare the three in terms of their side effects profile. We searched through PubMed and Google Scholar and pinpointed 13 articles aligned with our inclusion criteria: six articles utilized eptifibatide, four were related to abciximab, and three used tirofiban. In 85% of the cases, a severe drop in platelet count, reaching as low as 1000/μL, was reported.

Additionally, several other side effects were noted: one case documented multiple bruising spots appearing around the patient's body, two cases reported diffuse alveolar hemorrhage, and one case described a cardiac tamponade resulting from hemorrhagic pericarditis. Our study highlights the crucial significance of keeping a watchful eye on and comprehending the potential drawbacks linked to these medications in cardiovascular treatment. The necessity of researching these medications and their side effects is also evident, as this will significantly enhance the quality of treatment provided.

## Introduction and background

ST-elevation myocardial infarction (STEMI)

A substantial amount of evidence has emerged, underscoring the significance of prompt reperfusion therapy in the treatment of ST-elevation myocardial infarction. ST-Elevation Myocardial Infarction (STEMI) belongs to the acute coronary syndrome (ACS) group of diseases. It is a critical medical condition distinguished by the complete blockage of one or several coronary arteries responsible for supplying blood to the heart, resulting in myocardial necrosis. Clinical presentation included myocardial ischemia, electrocardiogram (EKG) alterations, and chest pain [1].

The rapid alteration in blood circulation can be attributed to various underlying reasons, namely the occurrence of plaque rupture, fissuring, or erosion, which subsequently leads to the formation of an obstructive thrombus [2]. Smoking, high blood pressure, high cholesterol, diabetes mellitus, genetics, and dyslipidemia are the most significant risk factors for STEMI [3].

The present definition of myocardial infarction (MI) necessitates the verification of myocardial ischemia injury by the presence of aberrant cardiac biomarkers [4].

Non-ST Elevation Myocardial Infarction (NSTEMI)

Non-ST elevation myocardial infarction ( NSTEMI ) is classified as a subset of acute coronary syndrome. It arises from an array of probable etiological factors, including cigarette dependency, sedentary lifestyle, hypertension, hyperlipidemia, diabetes mellitus, obesity, and familial history [5].

The individual experiences a sensation of pressure, like substernal pain. When the volume of the thrombus is inadequate to block the artery completely or only does so temporarily, the resulting reduction in blood supply to the afflicted myocardium is comparatively less severe or occurs intermittently. Under such conditions, it is common to observe myocardial necrosis, which an increase in cardiac-specific serum biomarkers such as troponin can identify. This clinical presentation is referred to as non-ST elevation myocardial infarction (NSTEMI) [6].

Epidemiology

Every year, more than seven million people in the world are diagnosed with Acute Coronary Syndrome [7]. In 2013, 116,793 people in the United States developed an MI, with 57% befall in men and 43% in women [1], with an average age of incidence in the first heart attack being 72 for women and 65.1 for men. Around 38% of patients who arrive at the hospital with ACS have a STEMI [1]. The statistics of 2013 demonstrated that one in three people died due to cardiovascular diseases in the United States [8]. However, the mortality due to MI has been declining because of new treatment strategies and better management [8].

Drugs used in STEMI patients

Certain classes of drugs play a role in reducing ischemic damage and relieving symptoms, which, in turn, may lead to improved outcomes in STEMI. One type is using thrombolytic agents such as alteplase and tenecteplase, which dissolve an obstructive clot within the coronary arteries [9]. Other common types are antiplatelet agents such as aspirin, and P2Y-12 receptor inhibitors like clopidogrel or ticagrelor are given to prevent further platelet aggregation and thrombus formation [10].

Subsequently speaking, beta-adrenergic blockers, such as metoprolol, mainly work to reduce myocardial oxygen demand, control the heart rate, and stabilize an infarcted heart [11]. For pain relief and vasodilation, sublingual or intravenous administration of nitroglycerin is utilized [12]. Lastly, glycoprotein IIb/IIIa inhibitors such as Abciximab, eptifibatide, and tirofiban primarily work to inhibit integrin-binding proteins responsible for platelet aggregation [13]. This review aims to further detail the description and implications of glycoprotein IIb/llla inhibitors in STEMI patients.

Drugs used in NSTEMI

A wide arsenal of drugs is indicated in the management of non-ST-segment elevation MI. They are categorized according to the nature of the desired outcome obtained by their functions.

The first group of drugs is the Anti-ischemic drugs. Nitroglycerin, a vasodilator, reduces myocardial oxygen demand by decreasing ventricular preload. β-Blockers decrease myocardial contractility and heart rate by preventing adrenergic stimulation of the myocardium [14]. Calcium channel blockers inhibit the contraction of both the myocardium and the vascular smooth muscle, reducing myocardial oxygen demand, causing coronary vasodilatation, and improving myocardial blood flow [15].

Angiotensin Converting Enzyme inhibitors are recommended because mortality rates were substantially reduced in the first 24 hours of initiating them [15]. Aldosterone antagonists (i.e., spironolactone, eplerenone) have been found to reduce morbidity and mortality in select patient populations, recommended after an ACS event for patients who are on therapeutic doses of an ACE inhibitor or ARB and β-blocker with an LVEF ≤40% and either symptomatic HF or diabetes mellitus [16]. Another group of drugs used are the anticoagulants. Low molecular weight Heparins (LMWHs) such as dalteparin, enoxaparin, and nadroparin are active against both factor Xa and factor IIa; therefore, they inhibit both the action and the generation of thrombin. Trials of oral anticoagulation have demonstrated the benefit of combining warfarin plus aspirin over aspirin alone, provided a sufficient degree of anticoagulation was achieved [14].

Antithrombotic drugs are another important class of drugs. These prevent the progression of existing and further development of newer clots in the coronary arteries. The first class of drugs is the antiplatelets. Aspirin blocks the synthesis of thromboxane A2, thereby diminishing platelet aggregation [15]. Prasugrel, Ticagrelor, and Vorapaxar are the newer P2Y12 receptor blockers [16]. GP IIb/IIIa Inhibitors (Abciximab, eptifibatide, and tirofiban) act by interrupting the final common pathway of fibrinogen-mediated cross-linkage of platelets [15].

GPIIb/IIIa inhibitors and their mechanism of action

Gp2b/3a inhibitors belong to a group of medications that hinder the attachment of fibrinogen to platelets [17]. They act by blocking the alpha and beta subunits found on the glycoprotein receptors of platelet membranes [17]. These are approved by the Food and Drug Administration (FDA) for acute coronary intervention. They play a vital role in situations with a considerable risk of blood clot formation [17-19]. They are administered via the intravenous route [17]. 

Eptifibatide hinders platelet aggregation by impeding the attachment of fibrinogen, von Willebrand factor, and other adhesive agents to these receptors. Eptifibatide demonstrates a dose-dependent inhibition of platelet aggregation, reversible upon discontinuation of the infusion. This reversal is attributed to the detachment of eptifibatide from the platelets [18]. Tirofiban acts as a reversible blocker of fibrinogen attached to these receptors, a crucial platelet surface receptor in platelet aggregation. Tirofiban demonstrates a dose and concentration-dependent inhibition of platelet aggregation [19]. Abciximab is derived from a Fab Antibody fragment derived from the chimeric human-murine monoclonal antibody 7E3. It works by reversibly binding to platelet receptors, preventing them from aggregating. This inhibits blood clot formation by blocking the attachment of specific molecules [20].

GPIIb/IIIa inhibitor uses & clinical guidelines

GPIIB/IIIA inhibitors are used to treat NSTEMI and STEMI [21]. In patients with acute STEMI, these inhibitors, like Abciximab are used in adjunct to percutaneous transluminal coronary angioplasty or stenting [22].

Current European Society of Cardiology guidelines about patients without ST-segment elevation myocardial infarction (NSTEMI) recommend concomitant use of GP inhibitors with dual antiplatelet therapy in patients undergoing high-risk PCI and without high bleeding risk as they have efficacy, rapid onset, and reversibility of action, absence of pharmacogenomic variability, pharmacoeconomic considerations and the possibility of intracoronary administration [23].

When the efficacy of the three drugs is compared, Eptifibatide is the most potent, while Abciximab has the most variable action, and Tirofiban has decreased efficacy in the treatment of STEMI and NSTEMI [24]. A considerable number of patients with ischemic heart disease may still be expected to require elective or emergency coronary artery bypass graft (CABG) after treatment with GP IIb-IIIa inhibitors [25]. 

GPIIb/IIIa inhibitors' side effects

GP 2b/3a inhibitors have an array of clinically relevant side effects that are important to acknowledge during the active management of a STEMI. During the first hours of administration, GP 2b/3a inhibitors can cause immune thrombocytopenia, which blocks the blood's ability to clot, leading to life-threatening bleeding if not recognized early when it occurs [26]. This resultant bleeding most often occurs over the femoral access sites but could also present itself as pulmonary alveolar, gastrointestinal, or genitourinary hemorrhage, and as a result of this bleeding, anemia can develop, requiring blood transfusion [27]. Eptifibatide, Abciximab, and tirofiban are known to cause this platelet dysfunction [28]. The interruption in the cascading sequence can cause severe refractory hypotension as an initial indication of reaction [29]. Lesser side effects of this drug type include bradycardia, vomiting, and administration site pain [30].

A gap in the literature and our review

Among the description of cases with side effects of glycoprotein IIb/IIIa inhibitors, the cases with incidences of thrombocytopenia have not been elucidated or thoroughly studied. It is imperative to consider this adverse effect as this can further lead to complications which, on certain occasions, have turned out to be fatal. In our extensive literature search, we have not encountered a case with thrombocytopenia being observed in patients who have been administered Orofiban and Roxifiban. Our review aims to go into further detail about the implications of these drugs (except Orofiban and Roxifiban) and shines a spotlight on this problem, hoping to bring out the importance of this void in the literature and suggest further research.

## Review

Methodology

Search Methods and Strategy

We conducted a comprehensive literature review to find appropriate articles by searching through PubMed and Google Scholar. Only peer-reviewed published case reports were included in our review. We searched from September 2023 through November 2023. The keywords used to find relevant articles were 'STEMI', 'nSTEMI', 'tirofiban', 'abciximab', 'eptifibatide', 'roxifiban', and 'orofiban'. We only included case reports as we wanted clinical/patient data on the side effects of these drugs. All differences in opinions were resolved by discussion with a senior author.

Data Screening and Eligibility

In doing so, we had 13 articles, 10 of which were on STEMI [31-40] and three on NSTEMI [41-43]. Two authors screened and assessed each article, and a senior author resolved any conflicts. We included articles that complied with our inclusion criteria. These criteria encompassed the following key factors: (1) articles indexed in PubMed, (2) study types limited to case reports and case series, (3) data derived from human subjects exclusively, (4) studies involving only adult participants (aged 18 and above), (5) reports on side effects linked to Gp2b/3a inhibitors, and (6) publications exclusively in the English language. Articles not meeting these criteria were excluded during this initial screening phase.

Full texts were obtained and subjected to a comprehensive review for the articles that passed the initial screening to ensure they adhered to all inclusion criteria. Studies that involved in vitro experiments and controlled trials were excluded from consideration as they may not directly provide information on clinical side effects. Studies reporting the use of the drugs in diseases other than STEMI and nSTEMI were also excluded. Additionally, pediatric and pregnant patients were excluded due to the potential confounding factors in these populations. Furthermore, studies conducted on animals or published in languages other than English were also excluded.

Data Collection and Analysis

We extracted data from 13 articles [31-43] to integrate into our review and systematically performed a comparative review. The studies were compiled and classified according to the first author, year of publication, the type of drug treatment, and whether the drugs were used in STEMI or nSTEMI. The database consisted of six articles on Eptifibatide, four on Abciximab, and three on Tirofiban.

Data were further collected in the specified categories when available: number of participants; features such as age and sex; comorbidities; reason for treating with a particular drug; dosage and duration of treatment provided; reported side effects; duration in which the side effects were noted; platelet count; interventions taken to treat the side effects; whether the causative drug was stopped or not; and the overall patient outcome. Two authors independently collected data and then cross-verified their findings with a senior author. Any disagreements were resolved through discussion with a senior author. Data was formulated using Microsoft Excel, and referencing was carried out using EndNote. This study did not require ethical or institutional review board (IRB) approval as data was sourced from pre-existing databases, and there was no direct involvement of humans or animals.

Results

Demographics

**Figure 1:**
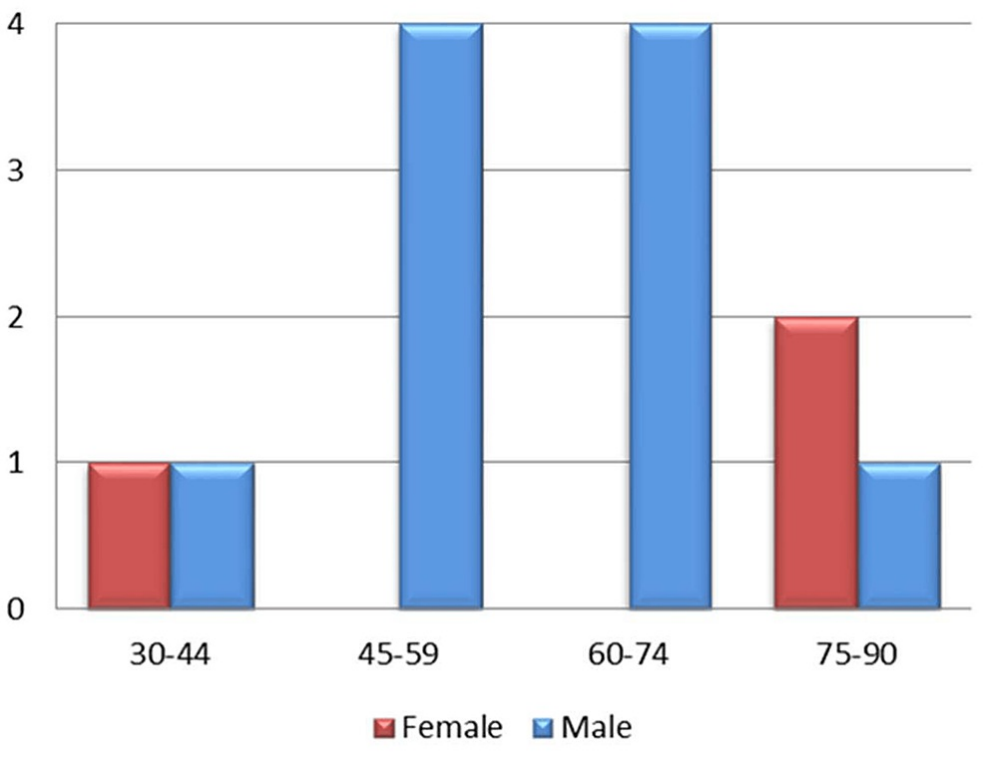
Age and Sex Distribution x-axis: Age Distribution y-axis: Number of Patients in our Included Records

We perused 13 records of case reports that summarized the effects of three different GpIIb/IIIa inhibitors - Abciximab, Eptifibatide, and Tirofiban (Table 1). The articles included treatment with these drugs for patients ranging from 34-84 years (Figure 1), with case reports published between 2002 and 2022.

**Table 1:** Summary of Records Included

Title of Paper	First Author	Year
Abciximab-induced acute profound thrombocytopenia post percutaneous coronary intervention	Todd Golden [41]	2017
Abciximab induced Alveolar hemorrhage treated with rescue extracorporeal membranous oxygenation	Andrew W. Choi [33]	2002
Acute Profound Thrombocytopenia Induced by Eptifibatide Causing Diffuse Alveolar Hemorrhage	Gregory Byrd [32]	2021
Delayed severe abciximab-induced thrombocytopenia: A case report	Łukasz Pia ˛tek [39]	2016
Eptifibatide-Induced Profound Thrombocytopenia After Percutaneous Intervention for Acute Coronary Syndrome.	Nirmanmoh Bhatia [31]	2017
Eptifibatide-Induced Severe Thrombocytopenia After ST-Elevation Myocardial Infarction (STEMI): A Case Report	Zaid Gheith [35]	2022
Eptifibatide-induced thrombocytopenia and thrombosis	Slava Epelman [34]	2006
Eptifibatide-induced thrombocytopenia	Marwan Refaat [43]	2007
Hemorrhagic pericarditis with cardiac tamponade after percutaneous coronary intervention associated with the use of abciximab	Su-Jin Moon [38]	2008
Left Main Stent Thrombosis Complicated by Eptifibatide-Induced Acute Thrombocytopenia	Eric H. Yang [40]	2011
Tirofiban-Induced Diffuse Alveolar Hemorrhage	Jincheng Guo [37]	2012
Tirofiban: A Rare Cause of Thrombocytopenia in a Patient Undergoing Percutaneous Coronary Intervention	Amit Gulati [36]	2021
A fatal complication of Tirofiban in diffuse alveolar hemorrhage	Erkan ilhan [42]	2010

Comorbidities considered in these patients were widely Diabetes Mellitus [32,40,42], hypertension [31,32,37-40,42], CAD [31,32,36,37,40], Prosthetic valve replacement [41], Liver cirrhosis [35]. Risk factors include smoking [31,36,38-40], alcohol use [31,35], and recreational drug use such as cocaine [31].

Reported cases had 10 male patients and three female patients with complaints of STEMI [31,33-39], NSTEMI [41,43], and PCI with angioplasty [31,32,37] in various scenarios. Abciximab was predominantly used in four cases aged 53-75 [33,38,39,41]. Eptifibatide [31,32,34,35,40,43] was used in six patients within the age range of 34-75 years. Tirofiban was used to treat three patients, 68-84 years of age [36,37,42]. Demographic data has been illustrated in Table 2.

**Table 2:** Demographic Data F: Female; M: Male; HTN: Hypertension; DM: Diabetes Mellitus; CAD: Coronary Artery Disease; VFib: Ventricular Fibrillation

GpIIb/IIIa INHIBITORS	NUMBER OF CASE REPORTS	AGE RANGES	SEX (F/M)		COMORBIDITIES (MOST COMMON)
ABCIXIMAB	4 [33,38,39,41]	53- 75 years	1 F	3 M	Prosthetic aortic valve, HTN
EPTIFIBATIDE	6 [31,32,34,35,40,43]	34-75 years	1 F	5 M	DM, HTN, CAD, VFib, Liver cirrhosis
TIROFIBAN	3 [36,37,42]	68-84 years	1 F	2 M	DM, HTN, CAD, Tuberculosis
TOTAL	13	34-84 years	3 F	10 M	

Abciximab

In the 13 case reports we reviewed, Abciximab was used in four of them [33,38,39,41]. Half of the patients had a significant history of smoking and hypertension [38,39]. In all the case reports, healthcare providers administered Abciximab during or after percutaneous coronary intervention due to an extensive thrombus burden. While all cases involved intravenous administration, one specified that Abciximab was directly applied to the coronary artery [41]. Remarkably, this specific case involved a patient with non-STEMI, unlike the other three that presented with myocardial infarction and ST-segment elevation.

There were variations in the dosage form used. In two cases, a single dose was administered [33,41]. The other two had an initial bolus given, followed by a 12-hour infusion [38,39].

All patients had an adequate platelet count upon admission. In two cases, thrombocytopenia was the sole side effect after drug administration, and it resolved without complications after discontinuing the medication and administering platelet transfusions [39,41]. In one case, a person's platelet count dropped significantly from 155,000/μL to 3,000/μL within nine hours after administration of the drug [41]. The other experienced a decrease in his count from 183,000/μL to 20,000/μL after 72 hours [39]. 

**Figure 2:**
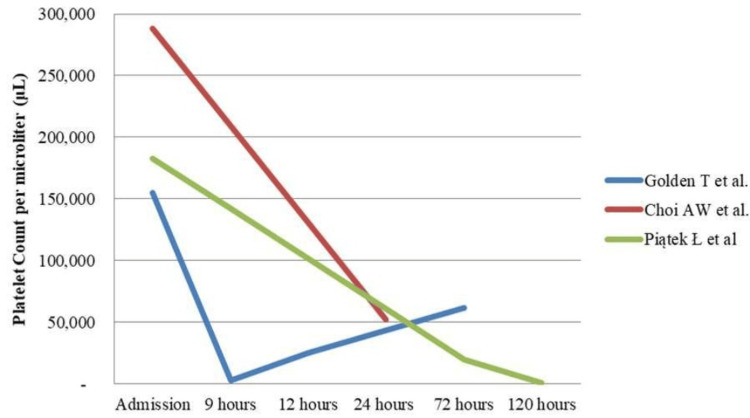
Trends in Platelet Counts From Three Different Studies [33, 39, 41]

Additionally, one patient, apart from developing thrombocytopenia (288,000/μL to 52,000/μL after 24 hours), also suffered from acute respiratory distress caused by diffuse alveolar hemorrhage only one hour after the procedure. This condition was effectively treated using veno-arterial extracorporeal membrane oxygenation [33]. See Figure 2 for an illustration of platelet count trends. Moreover, there was a case where a patient experienced pericarditis with cardiac tamponade 72 hours after receiving Abciximab. The treating team performed an emergency pericardiocentesis; platelet count was not mentioned [38].

Eptifibatide

Eptifibatide drug treatment was administered in patients undergoing cardiac catheterization in six studies, consisting of five STEMI cases [31,32,34,35,40] and one NSTEMI case (Table 3) [43]. Concomitant use of heparin during PCI was specified by five studies [31,34,35,40,43] except for one [32].

**Table 3:** Comparison of findings across the six case reports on Eptifibatide treatment

Author	Side effects	Duration of symptom onset	Baseline platelet count	Decrease in platelet count following eptifibatide therapy	Platelet count post-effective treatment	Discharge
Byrd G et al. [32]	Diffuse alveolar hemorrhage and profound thrombocytopenia	Within 12 hours	370,000	5,000	Not mentioned	9 days
Bhatia N et al. [31]	Left groin hematoma and profound thrombocytopenia	After 3 hours	180,000	7,000	123,000	1 day
Gheith et al. [35]	Gross hematuria, petechial rash, and profound thrombocytopenia	After 12 hours	103,000	3,000	35,000	72 hours
S. Epelman et al. [34]	Hypoxia, thrombosis (Deep venous thrombosis and diffuse thrombophlebitis in all four extremities) and profound thrombocytopenia	After 2 hours	200,000	8,000	Not mentioned	17 days
Eric H et al. [40]	Profound thrombocytopenia	Within 13 hours	276,000	12,000	50,000	12 days
M. Refaat et al. [43]	Profound thrombocytopenia	After 2 hours	298,000	6,000	Not mentioned	1 day

All six cases consistently reported profound thrombocytopenia [31,32,34,35,40,43]. The magnitude of this decrease varied, with the most substantial drop at 3000/μL [35] and the smallest drop at 12,000/mm3 [40]. Subsequently, this decrease in platelets led to various complications, including diffuse alveolar hemorrhage within 12 hours of drug therapy in one patient [32], profound hematoma formation after three hours reported by another [31], and hematuria and petechial rashes developing over 12 hours in yet another [35]. Interestingly, one particular study also raised concerns about eptifibatide potentially causing thrombosis where a patient developed deep venous thrombosis (DVT) and diffuse thrombophlebitis in all four extremities following drug therapy with eptifibatide [34].

Remarkably, the onset of these side effects surfaced within a 24-hour window. In three studies, the onset of symptoms surfaced around the 12-hour mark [32,35,40]. The side effects in the remaining three studies manifested around the two-hour mark [31,34,43].

An observation hinting at a potential trend is worth noting. Two STEMI cases featured patients reintroduced to eptifibatide therapy, with the initial treatment administered during their previous PCI, which took place five days earlier for one patient [32] and seven days earlier for another [40]. This pattern was reinforced by the NSTEMI study, where a patient was reintroduced to eptifibatide five days after the previous PCI and developed isolated profound thrombocytopenia after two hours [43]. In contrast, side effects also arose in three other patients, where two had their primary PCI [34,35], and one had undergone PCI and stenting a decade earlier [31].

Dosing patterns varied among the studies. In STEMI cases, two studies did not specify the dose of eptifibatide infusion administered [32,35]. Another two studies followed a similar regime of 180-μg/kg intravenous bolus and a 2-μg/kg/min infusion, suggesting a higher initial dose and faster infusion rate [34,40]. In contrast, one study administered 180 mg/kg initially, followed by 1 mcg/kg/min, suggesting a lower initial dose and a slower infusion rate in comparison [31]. In the NSTEMI case, the administered dosage was 180 μg/kg bolus for 10 mins followed by 2 μg/kg/h continuous infusion followed by another eptifibatide bolus of 180 μg/Kg [43].

To address side effects, eptifibatide was promptly stopped in all cases [31,32,34,35,40,43]. All patients with STEMI underwent platelet transfusion [31,32,34,35,40], and most stopped antiplatelet medications [32,34,35,40,43] except for one [31]. Additionally, heparin was discontinued in three patients [31,34,40], with two studies not specifying heparin treatment [32,35]. One patient warranted heparin reversal treatment with protamine sulfate 20 mg IV [31]. Ultimately, all patients recovered with their platelet levels rebounding and continually improving after receiving effective treatment. Patient discharge varied among studies, with the shortest being one day [31] and the longest extending to 17 days [34].

To rule out potential diagnoses such as heparin-induced thrombocytopenia and pseudo-thrombocytopenia, peripheral smear reviews were conducted in three studies [31,34,43], and the presence of heparin-platelets antibodies was checked in four [34,35,40,43]. One study acknowledged these possibilities but did not specify diagnostic testing [32].

Tirofiban

Tirofiban was administered in three cases among the 13 case reports reviewed [36,37,42]. In two case reports, Tirofiban was administered as an infusion [37,42], starting with an initial bolus dose followed by a maintenance dose of 0.15 mcg/kg/min. One started with a bolus of 25 mcg/kg [37], the other with half the bolus dose [42]. One case did not state the dosage that was used [36].

Following the administration of Tirofiban, most of them exhibited various side effects. One of the cases mentioned complaints of dry cough and displayed a drop in SpO2 from 95% to 60% after two hours and massive hemoptysis (Diffuse alveolar hemorrhage) after 2.5 hours [37]. In another case (Gulati A et al.), ecchymoses over the dorsal aspect of the right hand and wrist were noted [36]. One patient displayed symptoms of hemoptysis, dyspnea, and diffuse bilateral pulmonary infiltrates on chest radiography were observed [42]. 

Among the case reports, thrombocytopenia was noted in two cases [36,37]. One patient showed reduced platelets to 203/L and activated partial thromboplastin time (aPTT) of 71 seconds within two hours [37]. Another paper mentions that the patient's platelets were reduced from 208,000 to 4,000 after eight hours [36].

Following the appearance of the side effects in one patient, 40 mg IV furosemide and supplemental oxygen via a facemask were given for presumed pulmonary edema [37]. Then tirofiban was stopped, the patient was intubated due to increasing dyspnea, and bloody material was aspirated from the bronchi. He was recommenced with continuous carbazochrome and tranexamic acid infusion, and two units of packed red cells were transfused. His condition continued to deteriorate, and he died on postprocedural day seven [37]. In the other case report, a single unit of platelets was transfused, and the patient's platelet count improved [36]. For another patient, two units of erythrocyte transfusion, discontinuation of Tirofiban, and respiratory support were given. Still, bronchoscopy reported diffuse blood contamination of the bronchial tree without any active bleeding point. Then, unexpectedly, oxygen saturation worsened even with 100% FiO2, and bedside chest X-ray showed an increase of infiltrations in both lungs. Her respiratory condition did not improve, and she died on the third day [42]. 

Also noted in a particular case report post-STEMI, 300 mg of aspirin and 600 mg of clopidogrel were given [37]. During catheterization, (IABP) was inserted, and 5,000 units of unfractionated heparin were administered. In addition, the patient gives a significant history of STEMI, following which they underwent PTCA in LAD 4 years before the current presentation, Atelectasis in the left-lung, right-lung Emphysema, and left pulmonary bubble resections 30 years previously due to tuberculosis. Taking all of this into account, the thrombocytopenic effects of Tirofiban, along with the comorbidities mentioned above, could have contributed to the unfortunate fatal complications during the treatment [37].

Also to be considered is another case, which is the first tirofiban-related diffuse alveolar hemorrhage case caused by half of the recommended dose of tirofiban used in the setting of nSTEMI [42]. She also had a significant history of hypertension and diabetes. Interestingly, her platelet count did not seem to have deteriorated after administering and ceasing Tirofiban. The need for FiO2 disappeared on the second day, and ABG measurements showed progressive improvement. Hence, 75 mg clopidogrel and 2000 IU enoxaparin were prescribed twice daily but stopped immediately after the bronchoscopy report. On the third day, only 75 mg of clopidogrel was prescribed according to good medical conditions (normal ABG & no need for FiO2), but her condition deteriorated. Therefore, after a detailed analysis of the above events, Tirofiban could have induced the diffuse alveolar hemorrhage, which was then precipitated by the usage of other antiplatelet drugs, leading to fatal complications [42].

Eptifibatide, Tirofiban, Abciximab-A Comparison

In conducting a study including 13 case reports for the indication of GP2b/3a inhibitors for percutaneous coronary intervention in both STEMI and NSTEMI patients, eptifibatide was found to be used in 6 cases [31,32,34,35,40,43], tirofiban in 3 cases [36,37,42], and Abciximab in four cases [33,38,39,41]. All the patients included in the study had adequate amounts of platelets at the time of admission. All three drugs showed thrombocytopenia as a common side effect with a different degree of severity and complications.

Although the dose of administration of each drug for different patients was different, most of them had an initial IV bolus followed by an IV maintenance dose. Some had the initial bolus repeated. The dose of tirofiban was found to be less than that of eptifibatide. With Abciximab, apart from the IV bolus-maintenance regimen, one study specified the drug being directly applied to the coronary artery.

In two studies, eptifibatide was administered as 180-μg/kg intravenous bolus, followed by a 2-μg/kg/min infusion [34,40], whereas one study administered 180-μg/kg initially, followed by 1 mcg/kg/min infusion [41]. In two cases, Tirofiban had a maintenance dose of 0.15 mcg/kg/min [37,42] with an initial bolus of 25 mcg/kg in one study and half the bolus in the other. Abciximab was given as a single dose in two cases [33,41] and as an initial bolus followed by a 12-hour infusion in the other 2 cases [38,39]. Only eptifibatide had evidence of reintroduction of drug therapy after a previous PCI [32,40,43].

Thrombocytopenia was found in almost all the patients who were given these drugs, with the lowest platelet count noted to be 3000/μL with eptifibatide and 1000/μL with Abciximab [35,39] and 203/L with tirofiban [37]. Even though other side effects and complications due to thrombocytopenia were present with each drug, a fatal complication like diffuse alveolar hemorrhage was seen with all three drugs [32,33,37]. Diffuse alveolar hemorrhage developed within 12 hours with eptifibatide use, after 2.5 hours with tirofiban use, and after 1 hour with abciximab use.

On stopping the medicine and starting platelet transfusion and other supportive measures, all the patients' conditions on eptifibatide improved, and they were discharged. No fatality was reported with eptifibatide in the 13 cases we studied. With tirofiban use, even with discontinuing the medicine and providing effective treatment, two patients died [37,42] on days 3rd and 7th, respectively. Although one patient developed acute respiratory distress with abciximab use, the patient was successfully treated [33]. Table 4 summarises the comparison between the three drugs.

**Table 4:** Similarities and Differences Between GpIIB/IIIA Inhibitors

GP IIB/ IIIA inhibitors	ABCIXIMAB	EPTIFIBATIDE	TIROFIBAN
Route of administration	Intravenous / Intracoronary	Intravenous	Intravenous
Most reported side effect	Thrombocytopenia	Thrombocytopenia	Thrombocytopenia (worse)
Morbidity	Diffuse alveolar damage within 1 hour of drug administration	Diffuse alveolar damage within 12 hours of drug administration.	Diffuse alveolar damage within 2.5 hours of drug administration.
Systemic complications	Acute respiratory distress. Hemorrhagic pericarditis with cardiac tamponade.	-	-
Mortality	-	-	2 patients

Roxifiban and orbofiban are new glycoproteins in the group 2a/3b receptor inhibitors. Although these two medicines are linked to thrombocytopenia and bleeding, our extensive literature search found no case reports on these two drugs.

Discussion

Summary of our Findings

This passage provides a comprehensive review of 13 case reports involving the use of Gp2b/3a inhibitors (Abciximab, eptifibatide, and tirofiban) in patients aged 34-84, published between 2002-2022. Common comorbidities included diabetes mellitus, hypertension, coronary artery disease, prosthetic valve replacement, and liver cirrhosis, while risk factors encompassed smoking, alcohol, and cocaine use. Abciximab was administered in four cases, predominantly in STEMI patients, with varying dosages and thrombocytopenia as a side effect. Eptifibatide was used in six studies, primarily for STEMI cases, showing consistent significant thrombocytopenia, with one case reporting potential thrombosis. Discontinuations of Eptifibatide and platelet transfusions were common interventions, leading to patient recovery. The study highlights the crucial role of Gp2b/3a inhibitors in NSTEMI and STEMI treatment, with eptifibatide being the most potent, Abciximab showing variable action, and tirofiban exhibiting reduced efficacy.

Abciximab - Comparing and Contrasting our Findings with Those of Existing Literature

Abciximab (ABX) is a fragment of the monoclonal antibody 7E3 that binds the platelet receptor glycoprotein (GPIIb/IIIa) [44]. (ABX) has the strongest antiaggregant with a particular characteristic that predisposes patients to a higher incidence of both mild <100.000 cell/μL and severe < 50.000 cells/μL thrombocytopenia [39,45] by preventing the binding of fibrinogen and Von Willebrand Factor to GPIIb/IIIa receptor on activated platelets, showing a quick action, peak plasma concentration, and platelet inhibition achieved 10 minutes after IV injection [46]. The antiaggregant effect reached more than 90% 2 hours after the IV infusion. (ABX) is used as an antithrombotic therapy in Acute coronary syndrome and unstable angina who are not responding to conventional therapy also has a very well-known function to prevent acute ischemia, evidencing outcomes in patients followed by the realization of percutaneous coronary intervention [41]. Other studies showed that patients who received Abciximab for DVT developed a large retroperitoneal hematoma, likely due to severe thrombocytopenia [45]. However, there is a well-documented increase in bleeding and thrombocytopenia following the administration of two or more antiplatelet agents like Heparin and Abciximab [46].

Heparin-induced thrombocytopenia (HIT) should be considered as a differential diagnosis. Unlike HIT 2 caused by IgM, where thrombocytopenia occurs 5 to 10 days after exposure to heparin, causing mostly arterial and venous clots, platelet counts are higher than in abciximab-related thrombocytopenia [39], both are not associated with bleeding. This helps to differentiate from Abciximab, where we can find bleeding occurring frequently at the site of vascular access, petechial hemorrhages, epistaxis, subcutaneous hematoma, intracranial, digestive, retroperitoneal, hemorrhagic pericarditis, and urologic which are rare, and pulmonary hemorrhage are infrequent with high mortality of 20% and 50% in a small case series [33,41,47], lung conditions, such as COPD, pulmonary hypertension, and diseases with an increased pulmonary capillary wedge pressure, can be associated with an increased risk of pulmonary hemorrhage. Alveolar bleeding often requires Blood transfusions and platelets [47].

Treatment is controversial. Platelet transfusion and immediate cessation of Abciximab infusion and other drugs, like heparin, aspirin, and clopidogrel, appear to be the most effective treatment [39,48].

Eptifibatide - Comparing and Contrasting Our Findings with Those of Existing Literature

Eptifibatide is used most commonly under its brand name, Integrilin, and is one of several GP 2b/3a inhibitors acting as a therapeutic peptide [49]. Integrilin has played a critical role as part of a range of first-generation integrin antagonists, where it accompanies Abciximab, known as Reopro, and Tirofiban, known as Aggrastat [50]. Integrins have been targets of pharmacological interest for over 40 years; they are thought to play multiple roles in biochemical signaling between cells during varying ranges of human disease and health [51].

Integrilin is a peptide developed by imitating the venom of the southeastern pigmy rattlesnake. Compared to its counterparts, it has several benefits, such as a shorter half-life, specific receptor blocking, and rapid reversibility [52]. The venom of the pigmy rattlesnake is called barbourin, and its structure was found to be a specific inhibitor of GP 2b/3a inhibitors, but more importantly, its affinity was low for other known integrins [53]. Those same features found in Integrilin produced the desired pharmacokinetic and pharmacodynamic effect, which optimizes thrombosis inhibition with a short half-life and rapid onset alongside reversibility [53].

The qualities of the Eptifibatide produced an antithrombotic effect that was favorable in acute ischemic scenarios, most notably STEMI and nSTEMI events, and in these events, had reduced mortality significantly [54]. It is often used as adjunctive therapy with percutaneous coronary intervention (PCI) and, in large multicenter trials, is favorable concerning cost versus benefit [55].

Eptifibatide-induced thrombocytopenia (EIT) is a recognized complication and side effect of using the drug as an adjunct therapy in STEMI patients, which causes a profound loss of platelets in the blood [56]. EIT is thought to occur due to an immune response that clears or destroys sensitized platelets by a drug-dependent antibody [57]. Additional side effects of Eptifibatide administration are not suggestively prevalent [58]. Even in prolonged administration or overdose, where hypotension, bradycardia, and angioedema have been reported, they have been conservatively managed while waiting for renal clearance [58].

The identified case studies where Eptifibatide was used as an antithrombotic agent found similar side effects and impacts compared to the literature review. Effects in keeping with the review are EIT followed by various mechanisms of hemorrhage (such as petechia rash and alveolar hemorrhage). Notably, a new side effect not previously suggested is that of deep vein thrombosis and diffuse thrombophlebitis, which, given Eptifibadtide's interference with integrin signaling, could be responsible given its likely disruption of the clotting cascade [59].

In conclusion, Eptifibatide is a safe and cost-effective integrin antagonist that provides an excellent antithrombotic effect, reducing mortality in STEMI patients, and its side effects, namely EIT, are easily managed conservatively. Diffuse Thrombophlebitis could be a rarer and more problematic side effect than EIT, and more understanding of integrin influence is required to better our approach to STEMI management.

Tirofiban - Comparing and Contrasting Our Findings with Those of Existing Literature

The use of Tirofiban, one of the three glycoprotein IIb/IIIa inhibitors mentioned in this manuscript, is a tyrosine derivative that has a molecular weight of 495 kD, acts on fibrinogen by inhibiting it to the glycoproteins IIb/IIIa existing on the platelet. Overall, this leads to an inhibition of platelet aggregation and agents that promote platelet aggregation, such as adenosine diphosphate, collagen, epinephrine, and thrombin [60]. In ST-segment elevation myocardial infarction (STEMI) tirofiban has been a subject of interest and investigation in the realm of acute coronary syndromes. Along with other glycoprotein IIb/IIIa inhibitors such as Abciximab and eptifibatide, it has been studied for its potential to improve outcomes in this high-risk patient population [61].

Large-scale clinical trials such as the On-TIME 2 (Ongoing Tirofiban in Myocardial Infarction Evaluation 2) and the On-TIME 2 Extended follow-up have highlighted the benefits of the role of tirofiban in STEMI [62]. These studies have shown that early administration of tirofiban, either before or while undergoing a primary percutaneous coronary intervention (PCI) procedure, has reduced the occurrence of major adverse cardiovascular consequences, including death and recurrent MI events. It can also be noted in the cases reviewed also point out that Tirofiban administration post-PCI has reduced thrombosis.

Despite these positive findings, according to the literature, the main complication associated with tirofiban administration is diffuse bleeding (specifically, alveolar hemorrhage was mentioned in multiple case reports). Another rare complication is induced thrombocytopenia [36]. One exception was a patient who solely used tirofiban without undergoing a PCI, which resulted in no complications [63]. Careful patient selection and dosage adjustments are needed to reduce this risk.

In conclusion, tirofiban has shown promise in improving outcomes for STEMI patients, particularly when used in conjunction with PCI. However, its role may be reevaluated in the context of evolving treatment strategies and the availability of newer antiplatelet medications. Careful consideration of the risk-benefit profile is essential in its clinical application.

Other GP IIb/IIIa Receptor Antagonists

Roxifiban is an orally bioavailable GpIIb/IIIa receptor antagonist. Thrombocytopenia is a serious adverse event associated with this drug (approximately 2%) [64].

Orbofiban is another oral glycoprotein (GP) IIb/IIIa inhibitor. Though uncommon, orbofiban use was also associated with thrombocytopenia. Along with an increased risk of bleeding, orbofiban-induced thrombocytopenia also showed higher death rates, recurrent MI, intracranial hemorrhage, and major or severe bleeding [65]. 

Limitations

Recognizing the limitations inherent in a literature study is of utmost significance. Considering these obstacles, we can ensure that the existing knowledge deficit is effectively addressed. One constraint of our study is the absence of a meta-analysis, as its execution necessitates utilizing diverse tools, software proficiency, and other related resources. By their nature, in vitro investigations and controlled trials do not yield direct insights into the clinical side effects of medications. Consequently, these types of studies were not incorporated into the analysis. Finally, excluding pregnant and pediatric patients from our investigation was necessary due to possible confounding factors in these populations.

## Conclusions

Based on the study's results mentioned above, it was observed that the administration of Abciximab, eptifibatide, and tirofiban exhibited a significant correlation with severe thrombocytopenia and alveolar hemorrhage in patients. Life-threatening situations in patients following procedural administration should be assessed using a multidisciplinary approach and promptly addressed. This entails quick withdrawal of the medicine and implementation of other supportive measures. Not enough efforts have been made to consolidate research on the underlying mechanism responsible for developing thrombocytopenia or bleeding or on the two newer drugs (roxifiban, orbofiban). Furthermore, it is important to consider whether pharmacological evidence is available regarding the potential drug interaction that may occur when these medications are administered together with other antiplatelet agents, nevertheless, due to the extensive utilization of these medications in procedures such as percutaneous coronary intervention. To evaluate their efficacy and safety, it is imperative to conduct large-scale randomized control trials.
